# Organelle Inheritance Control of Mitotic Entry and Progression: Implications for Tissue Homeostasis and Disease

**DOI:** 10.3389/fcell.2019.00133

**Published:** 2019-07-23

**Authors:** Fabiola Mascanzoni, Inmaculada Ayala, Antonino Colanzi

**Affiliations:** Institute of Biochemistry and Cell Biology, National Research Council, Naples, Italy

**Keywords:** cell cycle, mitosis, golgi complex, organelles, mitotic spindle

## Abstract

The Golgi complex (GC), in addition to its well-known role in membrane traffic, is also actively involved in the regulation of mitotic entry and progression. In particular, during the G2 phase of the cell cycle, the Golgi ribbon is unlinked into isolated stacks. Importantly, this ribbon cleavage is required for G2/M transition, indicating that a “Golgi mitotic checkpoint” controls the correct segregation of this organelle. Then, during mitosis, the isolated Golgi stacks are disassembled, and this process is required for spindle formation. Moreover, recent evidence indicates that also proper mitotic segregation of other organelles, such as mitochondria, endosomes, and peroxisomes, is required for correct mitotic progression and/or spindle formation. Collectively, these observations imply that in addition to the control of chromosomes segregation, which is required to preserve the genetic information, the cells actively monitor the disassembly and redistribution of subcellular organelles in mitosis. Here, we provide an overview of the major structural reorganization of the GC and other organelles during G2/M transition and of their regulatory mechanisms, focusing on novel findings that have shed light on the basic processes that link organelle inheritance to mitotic progression and spindle formation, and discussing their implications for tissue homeostasis and diseases.

## Introduction

Entry into mitosis requires major cell reorganization to allow the proper inheritance of the genetic material between the daughter cells (Champion et al., 2017). The cells follow a specific and coordinated series of events to complete a successful division cycle. Mitosis is triggered by a complex regulatory circuit that controls the activation of the Cyclin-dependent kinase 1 (CDK1)/CyclinB complex, which is the master regulator of the mitotic onset (Nigg, 2001). Entry into mitosis and its proper completion are under the surveillance of checkpoints, which arrest the progression of the cell cycle if DNA damage or spindle failures are detected (Nigg, 2001). The irreversible commitment to mitotic entry is associated with a rapid and profound reorganization of cell shape. The cells become progressively round, centrosomes separate, the microtubules (MTs) organize the spindle apparatus, chromatin is condensed, and the nuclear envelope is disassembled. The latter event allows the spindle to capture and segregate the chromosomes (Champion et al., 2017).

Interestingly, it is now evident that also the proper reorganization and segregation of cellular organelles during mitosis is indispensable to ensure a correct cell division. Several studies have demonstrated that perturbations of the redistribution of specific organelles result in profound alterations of the cell division process (Jongsma et al., 2015), with potential consequences on tissue development and homeostasis. For example, epithelia are specialized animal tissues that form protective barriers lining the organs and the body. Each epithelium is characterized by a specific structural organization and cell composition. In response to constant turnover and environmental insults, epithelia maintain homeostasis through a tight balance of cell duplication, differentiation, and death (Tai et al., 2019). Moreover, cell divisions must also follow a predetermined orientation to preserve correct tissue architecture. Thus, errors in the control of the number and orientation of cell divisions can have detrimental consequences, compromising tissue development and/or function, and potentially leading to tumor progression (Ragkousi and Gibson, 2014).

Here, we review new findings related to the mechanisms underlying the mitotic segregation of subcellular organelles, and discuss how they impact on the proper mitotic progression, with an emphasis on those involved in the coordination of Golgi complex (GC) inheritance with the cell cycle.

## Preparation of Cell Entry Into Mitosis

**FIGURE 1:**
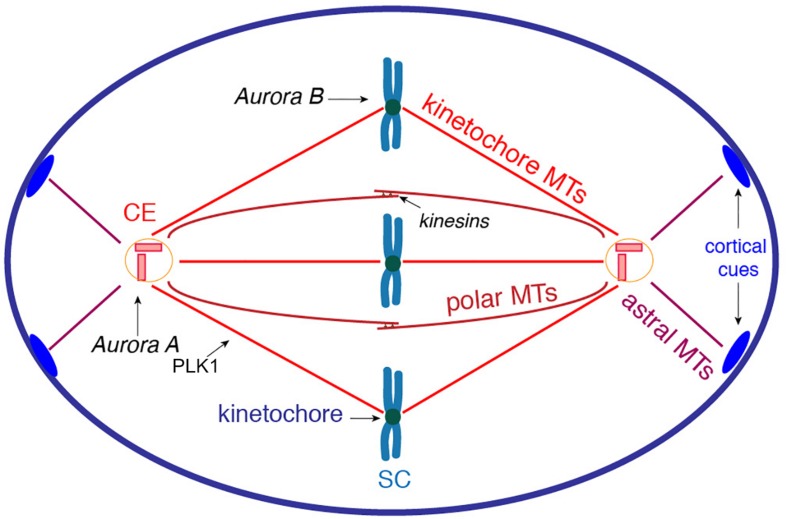
Schematic representation of spindle organization during mitosis. The centrosomes organize three types of MT fibers that are polymerized from the γ-TuRC associated to the PCM. The kinetochore MTs are directed toward the kinetochores to generate the traction forces required to separate the sister chromatids. The polar MTs form antiparallel spindle fibers; kinesin motors at the spindle midzone exert sliding forces to induce both separation and elongation of the spindle poles. The astral MTs are pulled toward specific cortical cues (blue ovals) to generate rotation forces required to reach proper spindle orientation. Key kinases involved in spindle formation: Aurora A associates with centrosomes and regulates centrosome maturation, mitotic entry and spindle elongation; Aurora B orchestrates chromosome-MT attachment and sister chromatid cohesion; and PLK1 regulates MT dynamics during spindle formation. CE, centrosome; SC, sister chromatids.

The primary event of cell division is the segregation of sister chromatids between the daughter cells. This essential task is achieved by the spindle apparatus, which is a very complex system that is organized by the centrosomes and composed of MTs and hundreds of regulatory proteins (Heald and Khodjakov, 2015). The recruitment of the γ-Tubulin Ring Complex (γ-TuRC) to the pericentrosomal region stabilizes MT minus ends and creates anchor points associated to the microtubule-organizing center (MTOC). The γ-TuRC act as a template for the polymerization of α/β tubulin heterodimers into MTs, which then undergo rapid growth and catastrophe phases (Petry and Vale, 2015; Petry, 2016). The centrosomes organize three types of MT fibers: kinetochore, polar, and astral MTs (Figure 1). The kinetochore MTs are directed toward the kinetochores and are responsible for the traction forces required to separate the sister chromatids. The polar MTs form antiparallel fibers that exert sliding forces mediated by kinesins, and thus, accomplish both separation and elongation of the spindle poles in mitosis. The astral MTs associate with specific domains of the cell cortex (Figure 1; Petry, 2016).

The assembly of the spindle is regulated by phosphorylation events. The kinase Aurora A associates with the centrosomes and is a crucial regulator of mitotic entry and centrosome maturation, which consists of the recruitment of proteins involved in MT nucleation and anchoring (Marumoto et al., 2002). Aurora B is a fundamental player in chromosome segregation because it controls the chromosome-microtubule attachment and sister chromatid cohesion (Hindriksen et al., 2017). Polo-like kinase 1 (PLK1) is involved in spindle maintenance by regulating MT dynamics through the phosphorylation of Microtubule-associated proteins (MAPs) and kinesin motors (Figure 1; Bruinsma et al., 2012). The concerted lengthening and shortening of the kinetochore, and of the polar spindle MTs, control the proper alignment of chromosomes at the spindle midzone (Heald and Khodjakov, 2015; Petry, 2016). The correct attachment of the chromosomes to the mitotic spindle is monitored by a spindle assembly checkpoint (SAC), which is activated in the case of incorrectly attached kinetochores, resulting in a delay of anaphase onset. The impairment of this checkpoint can lead to aneuploidy, aging and cancer progression (Lara-Gonzalez et al., 2012).

An additional crucial event during cell division is the orientation of the spindle apparatus, as it determines the proper orientation of the cell division axis (Lu and Johnston, 2013). After its formation, the spindle apparatus is subjected to a rotation process. The astral MTs associate with specific cues at the cell cortex, from where they are subjected to pulling forces (Figure 1). Thus, complex signaling pathways coordinate MT-mediated forces that are originated at the cell cortex and are applied on the spindle MTs (Lu and Johnston, 2013).

A critical preparatory step for spindle formation is the progressive cell reshaping from a flat to a spherical geometry that occurs during the G2/M transition (Champion et al., 2017). The rounding process becomes detectable during late prophase, but the preparatory steps begin during G2, when the focal adhesions (FA) undergo selective disassembly. This process is essential to achieve the progressive retraction of the cell margins and the formation of the actomyosin cortex, which are necessary for the cell rounding in metaphase (Cadart et al., 2014; Champion et al., 2017). Cell rounding is necessary to create a symmetric cell organization that allows the kinetochore MTs to capture the chromosomes. In fact, a reduced rounding can cause the scattering of chromosomes, which decreases the probability of being captured by the kinetochore MTs. Besides, an asymmetric cell geometry can induce spindle deformations, resulting in the splitting of the spindle poles and formation of acentriolar spindles, thus increasing the likelihood of multipolar cell division and aneuploidy. Therefore, alterations of mitotic rounding can affect spindle morphology, chromosome segregation, and timely mitotic progression (Champion et al., 2017). In addition, the geometry of the cell during mitosis also influences the orientation of the spindle and, as a consequence, the direction of the cell division axis, and thus tissue morphogenesis and differentiation (Morin and Bellaiche, 2011). Collectively, these findings emphasize that correct cell geometry and the controlled redistribution of cell components are crucial for the daughter cells fate, and errors in the process can lead to the development of diseases (Lancaster et al., 2013).

Entry into mitosis is also accompanied by an extensive reorganization of subcellular organelles, which undergo stereotyped reorganization of the structure and localization (Champion et al., 2017). For instance, during G2 the centrosomes move from the perinuclear area to the center of the nucleus in order to be disengaged and separated (Sutterlin and Colanzi, 2010). The reorganization of organelle morphology can range from the full disassembly of the GC to the subtle modifications of the endosomes (Lowe and Barr, 2007; Jongsma et al., 2015). In the next paragraphs, we will summarize the current knowledge about the mitotic fate of the intracellular organelles, with a focus on the GC, and referring to several recent reviews for more mechanistic details.

## The Golgi Complex

### Structural Reorganization of the Golgi Complex During Mitosis

The GC has a pivotal role in the secretory pathway, as it is involved in the modification and sorting of cargoes (Wei and Seemann, 2010). In mammalian cells, the GC is characterized by a ribbon structure, which is composed of several polarized stacks of cisternae that are laterally connected by tubules (Lowe, 2011). The structure of the GC is maintained by the Golgi Reassembly Stacking Protein of 55 and 65 kDa (GRASP55 and GRASP65) (Zhang and Wang, 2015) and by the members of the golgin family (Gillingham and Munro, 2016). All these proteins act as membrane tethers and concur in the stacking of the cisternae and in directing the formation of the membranous tubules connecting the stacks (Xiang and Wang, 2010; Witkos and Lowe, 2015). The perinuclear position of the GC is maintained through the association with MTs (Maia et al., 2013).

**FIGURE 2:**
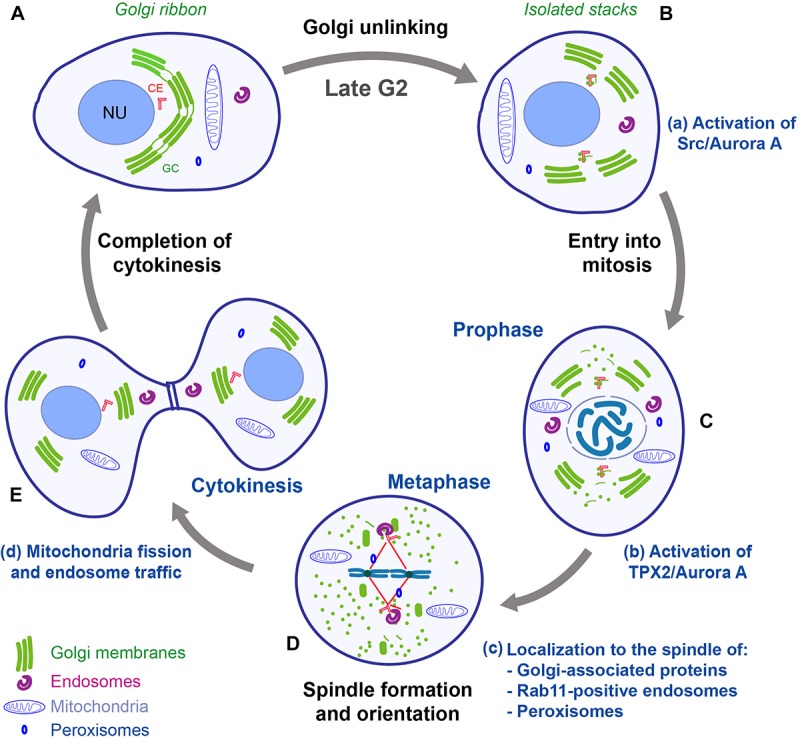
Schematic representation of mitotic redistribution and inheritance of organelles and their connection with the cell cycle. In mammalian cells, during mitosis, the organelles are subjected to complex structural reorganizations. **(A,B)** During G2, the Golgi ribbon is converted into isolated stacks, and the centrosomes are separated. Golgi unlinking activates a Src/Aurora A signaling pathway that is required for entry into mitosis and centrosome maturation **(a)**. **(C)** During prophase, the GC is disassembled into dispersed vesicles and vesicular/tubular clusters; the endosomes, mitochondria and peroxisomes are separated and evenly redistributed in the cell. The disassembly of Golgi stacks allows the release of proteins and the activation of a TPX2/Aurora A pathway that concur to aid the formation of the spindle **(b)**. **(D)** During metaphase, several organelle-based protein machineries (e.g., GM130, Rab11 endosomes, and peroxisomes) **(c)** contribute to organize the formation of the spindle and to achieve correct chromosome alignment at the metaphasic plate. **(E)** After the formation of the cleavage furrow, the cells are ready to complete the cytokinesis process, which requires mitochondria fission and endosome traffic **(d)**. Impairment of one of the membrane-based processes can cause several defects in correct completion of mitosis, with potential repercussions on tissue homeostasis and diseases development. CE, centrosome; GC, Golgi complex; NU, nucleus. Adapted with permission from Ayala and Colanzi (2017).

During late G2, the Golgi ribbon is divided into individual stacks (unlinking) (Figures 2A,B). The pro-ribbon role of the GRASPs is inhibited by phosphorylation. The basic regulatory elements have been identified, and they include the kinase PKD that leads to a RAF1/MEK1/ERK1-mediated phosphorylation of GRASP55 (Valente and Colanzi, 2015). In addition, a major inducer of ribbon unlinking is phosphorylation of GRASP65 by JNK2 and PLK1 (Acharya et al., 1998; Colanzi et al., 2000; Feinstein and Linstedt, 2007; Sengupta and Linstedt, 2010; Tang and Wang, 2013; Rabouille and Linstedt, 2016). Moreover, Golgi unlinking requires the fission-inducing protein BARS to cleave the tubules connecting the stacks (Hidalgo Carcedo et al., 2004; Colanzi et al., 2007). For more mechanistic details the reader is referred to several reviews (Lowe and Barr, 2007; Corda et al., 2012; Tang and Wang, 2013; Ayala and Colanzi, 2017; Wei and Seemann, 2017). During prophase (Figure 2C), the activation of CDK1 leads to the phosphorylation of additional sites on golgins and GRASPs, resulting in complete inhibition of the membrane tethering processes. Furthermore, CDK1 also induces the phosphorylation of adaptor proteins involved in membrane fusion, such as p47/VCIP135 and p37, causing inhibition of membrane fusion events (Uchiyama et al., 2003; Kaneko et al., 2010; Tang and Wang, 2013; Totsukawa et al., 2013). An additional contribution to the inhibition of the membrane fusion machineries is provided by the HACE1-mediated monoubiquitination of the SNARE protein syntaxin 5 (Huang et al., 2016). As a result, the Golgi membranes are consumed by an extensive vesiculation during metaphase, when they become dispersed into vesicular/tubular clusters, with some intermediate compartment (IC) and Golgi proteins redistributed into the Endoplasmic Reticulum (ER) (Figure 2D; Saraste and Marie, 2018). Then, during telophase and cytokinesis, the dispersed Golgi proteins and membranes are gradually reassembled into a GC in each of the daughter cells (Figure 2E; Shorter and Warren, 2002; Altan-Bonnet et al., 2004; Colanzi and Corda, 2007).

Importantly, the disassembly of the GC is also a requirement for mitotic entry. Indeed, blocking the unlinking step induces a potent G2 block of the cell cycle, pointing out that a mitotic “Golgi checkpoint” oversees the correct premitotic cleavage of the GC (Sutterlin et al., 2002; Hidalgo Carcedo et al., 2004). Although the existence of a mechanism that controls the correct partitioning of the organelle could be surprising, recent findings are starting to reveal the general framework, and the evidence suggests that a physical/functional connection of the GC with the centrosomes and the MT network is at the basis of this novel checkpoint.

### Role of the Centrosome in the Localization and Structure of the Golgi Complex in Interphase

It is already known that the structure and localization of the GC can be modulated by the centrosome through multiple mechanisms (Sutterlin and Colanzi, 2010; Rios, 2014). The centrosome is composed of two centrioles enclosed by pericentriolar material (PCM), which consists of a thick shell of multiprotein complexes. In most cell types, the centrosome represents the major MTOC, and is involved in the formation of radial MT fibers (Sanchez and Feldman, 2017; Muroyama and Lechler, 2017). The template for MT nucleation is the γ-TuRC, which is recruited to the centrosome. The centrosome is positioned at the cell center, close to the nucleus. Following a polarization stimulus, it is reoriented in the direction of the leading edge of the cell (Pouthas et al., 2008).

The centrosomal MTs form radial fibers that guide the positioning of the Golgi membranes toward the cell center thanks to dynein, which is a minus end-directed motor complex (Rios, 2014) that is recruited at the GC by Golgin160 (Yadav et al., 2012). Also, the actin cytoskeleton contributes to the maintenance of the ribbon, as it forms tracks for actin-based motors (Valderrama et al., 1998). Besides, a subset of MTs is nucleated at the GC (Chabin-Brion et al., 2001). Nucleation of MTs from the GC is driven by a multiprotein complex that is organized by the scaffold protein AKAP-450, which is recruited at the *cis*-Golgi by GM130 (Zhu and Kaverina, 2013). The newly nucleated MTs are then stabilized and anchored at the *trans-*Golgi network (TGN) through the MT binding proteins CLIP-associated proteins 1 and 2 (CLASP1/2), due to their ability to stabilize MT plus ends by suppressing MT catastrophes (Efimov et al., 2007; Wu et al., 2016). The simultaneous knockdown of both CLASPs reduces MT nucleation at the GC and causes mitotic defects, such as multipolar spindle formation, and consequent cytokinesis failure (Table 1; Mimori-Kiyosue et al., 2005; Lansbergen et al., 2006; Pereira et al., 2006).

**TABLE 1:** Golgi located proteins involved in spindle formation.

**Protein**	**Definition**	**Phenotype after depletion**	**References**
SAC1	Suppressor of actin mutations 1-like protein	Golgi fragmentation and spindle disorganization	Liu et al., 2008
RINT-1	RAD50-interacting protein 1	Golgi disruption and multipolar spindle structures	Lin et al., 2007
Tankyrase-1	Poly (ADP-ribose) polymerases	Spindle bipolarity and other morphological defects.	Chang et al., 2005
p115	General vesicular transport factor p115	Fragmented Golgi and unstable spindle formation	Radulescu et al., 2011
GM130	Golgin subfamily A member 2	Multipolar spindles	Kodani and Sutterlin, 2008
GRASP65	Golgi reassembly stacking protein	Multiple aberrant spindles, metaphase arrest, and cell death	Sutterlin et al., 2005
CLASP1-CLASP2	Cytoplasmic linker-associated protein 1 and 2	Short pole-to-pole distance in bipolar spindle and multipolar spindle formation	Mimori-Kiyosue et al., 2005
Miki	Mitotic kinetics regulator	Pseudometaphase and multinucleated cells with micronuclei	Ozaki et al., 2012

The GC-based MT nucleation is crucial not only for the structural integrity of the GC, but also for the formation of asymmetric MTs that are essential for the orientation of the GC toward the leading edge during migration (Vinogradova et al., 2009; Kaverina and Straube, 2011; Wu and Akhmanova, 2017), and for the polarized delivery of cargoes (Miller et al., 2009; Sanders and Kaverina, 2015). Yet, the significance of the GC-centrosome proximity and of the ribbon organization are not completely understood. In particular, the knockdown of the golgin GMAP210 or Golgin160 induces the unlinking into separated stacks, which are still able to transport cargoes to the cell surface but that become unable to direct the secretion toward specific domains of the plasma membrane (PM) at the leading edge. As a result, the directional persistence of cell migration is reduced (Yadav et al., 2009). In agreement with these observations, depletion of GM130 using siRNA in UO2S or HeLa cells results in ribbon unlinking and reduced efficiency of cell migration (Kodani and Sutterlin, 2008). Additionally, experiments based on the expression of various N-terminal fragments of AKAP-450 led to the conclusion that the proximity of the GC to the centrosome, but not the presence of an intact ribbon, is the crucial factor for optimal directional cell migration (Hurtado et al., 2011). However, experiments based on RPE1 cells in which GM130 was knocked out led to the conclusion that a close association of the GC with the centrosome is not required for cell migration or protein transport (Tormanen et al., 2019). Therefore, more investigations are needed to better understand the functional consequences of perturbations of the GC structure, or of its proximity to the centrosome.

### Physical and Functional Relationships Between the Golgi Complex and the Centrosome During Mitosis

The structural reorganization of the GC during the cell cycle appears to be coordinated with those of the centrosome (Sutterlin and Colanzi, 2010). The centrosomes are duplicated during S-phase; then, during G2, they are pulled apart, in coincidence with the severing of the Golgi ribbon (Figure 2B; Persico et al., 2010). Thus, the GC is segregated into two groups of stacks, each of which is localized in proximity to a separated centrosome (Figure 2C). Furthermore, during this phase, the membranes of the IC remain closely associated with the centrosomes and become detached from the bulk of the GC, suggesting that the IC maintains its identity during mitosis and provides an intermediate station for Golgi dispersal (Marie et al., 2012; Saraste and Marie, 2018). In concomitance with these events, the composition and size of the PCM material are profoundly modified, involving the recruitment of other components, like Cep192/SPD-2, PCNT/PLP, and Cep215/Cnn, in a process that is defined as “centrosome maturation”(Joukov et al., 2014). The “mature” centrosomes reach their final position and orientation in metaphase, when they direct MT nucleation for the formation of the spindle, which is fundamental for correct segregation of the chromosomes into the daughter cells (Meraldi and Nigg, 2002). Defects in assembly and duplication of the centrosomes, and the consequent problems in MT nucleation, are the primary cause of the formation of aberrant spindles. In support of a functional GC-centrosome relationship, the G2-specific Golgi ribbon unlinking acts as a controller of the centrosomal recruitment of Aurora A (Persico et al., 2010), which is a major regulator of G2/M transition, centrosome maturation, and spindle formation (Figures 2B,C; Marumoto et al., 2002). In particular, Barretta et al. (2016) demonstrated that upon unlinking, Src is activated at the GC, then Src interacts with Aurora A and phosphorylates the residue Y148, increasing the kinase activity of Aurora A, which then is recruited to the centrosomes to induce their maturation (Figure 2a). Aurora A is a pivotal switch of spindle formation, as its inhibition or ablation causes formation of multipolar and/or fragmented spindles (Marumoto et al., 2002; Hoar et al., 2007; Malumbres and Perez de Castro, 2014). An additional line of evidence of the correlation between mitotic Golgi disassembly and centrosome-based functions, has been revealed thanks to an assay designed to prevent the disassembly of the Golgi stacks during mitosis through the controlled formation of 3,3′-diaminobenzidine (DAB) polymers in the Golgi lumen (Guizzunti and Seemann, 2016). Cells containing DAB polymers in the Golgi stacks entered into mitosis normally, but they arrested in metaphase with intact Golgi clusters associated with monopolar spindles, which caused SAC activation. Artificial disassembly of the GC relieved this block, suggesting that the disassembly of the Golgi stacks is required for progression through mitosis (Guizzunti and Seemann, 2016; Wei and Seemann, 2017).

In addition, several reports have shown a direct role of Golgi matrix proteins in assisting spindle formation. For example, the N-terminal domain of GM130 includes a nuclear localization signal (NLS) that has been shown to be essential for proper spindle assembly (Wei et al., 2015). During interphase, the NLS is masked by the interaction with the Golgi matrix protein p115. CDK1-mediated phosphorylation of GM130 dissociates p115 from GM130, and this triggers a crucial pathway of mitotic disassembly of the Golgi stacks (Nakamura et al., 1997; Nakamura, 2010; Wei et al., 2015), as it unmasks the NLS, which becomes able to bind and sequester at the Golgi the nuclear pore component importin-α. Of note, before mitosis onset, importin-α is bound to the spindle assembly factor TPX2 (Figure 2b), keeping this protein inactive. Accordingly, as a consequence of the binding of importin-α to GM130, TPX2 becomes free to interact with Aurora A, resulting in the increase of its kinase activity and local stimulation of MT nucleation required for the assembly of the spindle. Furthermore, once the spindle fibers are formed, they become “stabilized” by GM130 (Figure 2c), which directly binds and bundles MTs, thus linking Golgi membranes to the spindle (Figures 2C,D; Wei et al., 2015; Wei and Seemann, 2017). Probably correlated to this function, depletion of GM130 causes the formation of over duplicated centrosomes and multipolar spindles during mitosis (Table 1), resulting in metaphase arrest and cell death (Kodani and Sutterlin, 2008).

To further support the functional connection of the GC with the spindle, several reports have shown evidence of Golgi-associated proteins that influence spindle formation and mitotic progression. In this regard, the GM130 interactor p115 becomes associated with the mitotic spindle throughout mitosis. A specific armadillo-like fold of the N-terminus of p115 was responsible for its interaction with γ-tubulin and centrosomal targeting. Strikingly, p115 depletion causes spindle abnormalities, chromosome defects, and cytokinesis failure (Table 1; Radulescu et al., 2011). Also, the other GM130 interactor, GRASP65, has been endowed with mitosis-specific roles. Indeed, GRASP65-depleted cells show multiple disorganized and non-functional spindle asters (Table 1), indicating that GRASP65 regulates MT dynamics during entry into mitosis (Sutterlin et al., 2005). The list of Golgi-associated proteins with roles in spindle formation is not limited to the GM130-based protein complex. For instance, depletion of the Golgi-associated phosphoinositide phosphatase SAC1 causes perturbations of Golgi architecture and spindle abnormalities (Table 1; Liu et al., 2008). In addition, tankyrase-1 is an ADP-ribosyltransferase that is associated with the Golgi in interphase, and relocates to the spindle poles during mitosis. Its depletion causes mitotic arrest with abnormal chromosome segregation, bipolar spindle formation, and failure of telomere separation (Table 1; Chang et al., 2005). Also, the tankyrase-1 substrate Miki translocates from the GC to the centrosomes during the late G2/M phase. Depletion of Miki induces a pseudometaphase state that leads to the formation of multinucleated cells (Table 1; Ozaki et al., 2012). Another Golgi-associated protein implicated in cell cycle control is the Rad50-interacting protein RINT-1, whose depletion causes partial Golgi fragmentation, centrosome amplification during interphase, and increased formation of multiple spindle poles that culminate in frequent chromosome missegregation (Table 1; Lin et al., 2007).

More in general, the spindle recruits and directs the inheritance of Golgi matrix proteins that are involved in the formation of the Golgi ribbon, while a minimal set of proteins and membranes sufficient to reassemble functional Golgi stacks are inherited independently of the spindle (Wei and Seemann, 2009). It could be speculated that the Golgi matrix proteins recruited by the spindle are not simple passengers, but acquire different mitosis-specific functions. In support of this possibility, during mitosis, the small GTPase Arf1 becomes inactive and dissociates from the Golgi membranes (Altan-Bonnet et al., 2003), and this correlates with the dispersal of several peripheral Golgi proteins. If Arf1 is artificially kept active, Golgi membranes do not fragment, and the peripheral proteins remain associated with the GC throughout mitosis. These cells enter mitosis, but exhibit gross defects in chromosome segregation and cytokinetic furrow formation, resulting in multinucleation (Altan-Bonnet et al., 2003).

Thus, there is a substantial amount of evidence to conclude that an active functional interplay between the GC and the centrosome is crucial for spindle formation and, hence, for accurate segregation of the genetic material.

## Structural Reorganization of Other Organelles During Mitosis

### Endoplasmic Reticulum

The ER is a large continuous membranous organelle that is responsible for the synthesis of the majority of the integral membrane proteins and lipids. It comprises three different domains: the smooth ER (SER), the rough ER (RER), and the nuclear envelope. The ER constitutes a vast network of cisternae and tubules spread across the cytosol, and establishes contacts with several subcellular compartments (Phillips and Voeltz, 2016).

The ER undergoes marked structural modifications during mitosis. Specifically, the cisternae are transformed into mixed populations of tubules, the extent of which varies among cell lines (Puhka et al., 2007, 2012). In addition, during late prophase, the nuclear envelope is disassembled and its membranes reabsorbed into the bulk of the ER to expose the chromatin to the spindle apparatus. In prometaphase, the ER is split into two large pools of membranes that maintain continuity throughout mitosis (Lu et al., 2009; Schwarz and Blower, 2016). During anaphase and telophase, after chromosomal segregation, the nuclear envelope reassembles, and this marks the beginning of the reorganization of the ER compartment, although the underlying molecular mechanisms are poorly understood (Schwarz and Blower, 2016).

The ER is also a major hub for intracellular organization and signaling (Jongsma et al., 2015; Schwarz and Blower, 2016). Indeed, throughout interphase it forms multiple contact sites (CS) with the PM, endosomes, GC, and cytoskeleton (Phillips and Voeltz, 2016). Membrane CSs have crucial functions in inter-organelle signaling and lipid transfer. Considering its role as a major organizing compartment, it is likely that the inheritance of the ER is regulated by yet unknown control mechanisms. Recently, it has been shown that the ER–PM CSs undergo significant changes in morphology and function during mitosis (Yu et al., 2019). One of the major functions of CSs is to control Ca^2+^ signaling through the store-operated Ca^2+^ entry (SOCE), which depends on the integrity of ER–PM CS to allow contact between the proteins STIM1 and Orai1 (Yu et al., 2019). During mitosis, the density of these specific CSs is decreased. Also, the average distance between the PM and the closest ER in mitosis is increased. Therefore, the down-regulation of ER–PM junctions in mitosis induces SOCE inhibition by preventing the interaction of STIM1 with Orai1. This inhibition could affect not only Ca^2+^ signaling but also lipid metabolism and membrane structure (Yu et al., 2019).

### Endosomes

The membranous endocytic system mediates the traffic of lipids, proteins, and other molecules among various intracellular locations. These features have an essential impact on signal transduction and nutrient acquisition. Even if the various cellular functions of the endolysosomal system are extensively investigated, the mechanisms of endosome inheritance are marginally known. The current view is that endosomes and lysosomes remain intact during mitosis, and that during cytokinesis these organelles accumulate in the proximity of the MTOC (Bergeland et al., 2001; Jongsma et al., 2015). Despite this limited knowledge, an interesting aspect is that during prophase, MT-dependent motors induce the clustering of Rab11-positive endosomes around the centrosomes (Figures 2C,c). This localization is important to prevent their transport to the PM and to bring MT-nucleating proteins to the centrosome (Hehnly and Doxsey, 2014). For this reason, Rab11 relocation to the centrosome is necessary for the formation of a functional and properly oriented mitotic spindle. The clustered Rab11 endosomes are then segregated by the mitotic spindles between the daughter cells (Figures 2D,E; Hehnly and Doxsey, 2014). Moreover, in line with the proposed role of membrane reservoir in mitotic cells, during anaphase, the recycling endosomes are transported toward the cleavage furrow (Figure 2E; Bergeland et al., 2001), where they are believed to provide the membranes required to complete the cytokinesis (Simon and Prekeris, 2008).

### Peroxisomes

The peroxisomes are organelles involved in fatty acid and energy metabolism. During prophase, they are associated with the MT network and remain clustered around the spindle poles (Figures 2C,D,c). Throughout telophase, they are repositioned around the reforming nucleus of each daughter cell (Figure 2E; Hettema and Motley, 2009). Importantly, an intriguing connection of peroxisome inheritance with tissue development has been demonstrated. The peroxisome-associated protein PEX11b has been found to be essential for the differentiation of the skin, which undergoes a continuous renewal that is required to ensure a healthy tissue turnover (Asare et al., 2017). Specifically, the peroxisomes of PEX11b-depleted cells are functional, indicating a marginal role of this protein for the most classical peroxisome roles. However, while in control cells the peroxisomes localize at the spindle poles during mitosis, in PEX11b knockdown cells they fail to localize properly, resulting in a mitotic delay and SAC activation (Asare et al., 2017). Furthermore, PEX11b deficiency is associated to uncontrolled rotations of the spindle, which should normally be oriented perpendicularly to the basal membrane. The localization of peroxisomes at the spindle poles is the crucial factor, as their artificial relocalization to the cell cortex, or the spindle midzone, is sufficient to cause the alterations of spindle orientation. Importantly, in mouse embryos that are knockdown of PEX11b, the inability of the basal stem cells to orient their spindle perpendicularly to the basal membrane also led to alterations of their differentiation. These perturbations had severe effects, as the epidermis showed hyperproliferation and increased expression of terminal differentiation markers in basal cells, which is a feature typically associated with cancer (Asare et al., 2017). Thus, these data further support the notion that correct organelle inheritance/positioning is crucial for spindle formation and alignment, and they also revealed for the first time that these defects can have direct consequence on the development and maintenance of a stratified epithelium, where spindle orientation and cell differentiation are critical factors for establishing tissue structure and maintain homeostasis.

### Mitochondria

Mitochondria are the energy factories of cells and are characterized by the presence of a double membrane. The inner membrane is folded into numerous cristae, which increase the surface area, and contain circular DNA together with the protein components needed for transcription and translation (Friedman and Nunnari, 2014; Suarez-Rivero et al., 2016). Mitochondria are also major stores of Ca^2+^ ions. During cell proliferation, the mitochondria have to be segregated into daughter cells (Friedman and Nunnari, 2014). Upon entry into mitosis, the mitochondrial network is cleaved into fragments that are uniformly dispersed within the cytoplasm by an ordered mechanism of inheritance mediated by MTs. During cytokinesis, the mitochondria are recruited to the cleavage furrow, where they remain localized until completion of cell abscission (Figures 2D,d; Lawrence and Mandato, 2013a,b).

An important feature of this organelle is that it undergoes a dynamic equilibrium of fusion and fission processes. The fusion of mitochondria is operated by the dynamin-like GTPases mitofusins 1 and 2 (Mfn1 and Mfn2), and optic atrophy 1 (Opa1). Mfn1 and Mfn2 are localized on the mitochondrial outer membrane, while Opa1 resides on the inner membrane (Friedman and Nunnari, 2014). The fission process is mediated by the dynamin-related protein 1 (DRP1). Knockout of any of these proteins is embryonic lethal in mice (Friedman and Nunnari, 2014; Suarez-Rivero et al., 2016). During mitosis, Aurora A phosphorylates the small Ras-like GTPase RALA, which localizes to mitochondria and triggers the formation of a complex with RALBP1 and CDK1/CyclinB, inducing the phosphorylation of DRP1 to stimulate mitochondrial fission (Kashatus and Counter, 2011). The knockdown of either RALA or RALBP1 leads to the inhibition of mitochondrial division. As a result, the cells become unable to evenly distribute mitochondria between the daughter cells, resulting in cytokinesis defects (Kashatus et al., 2011). Interestingly, experiments performed in mammalian stem-like cells revealed that they have the capability of asymmetrically sorting young and old mitochondria. The daughter cell that maintains stem cell features is the one that receives most of the new mitochondria (Katajisto et al., 2015). This is important for maintaining stemness, as the block of mitochondrial fission impedes the asymmetric distribution of mitochondria, and results in loss of stem cell properties in the daughter cells (Katajisto et al., 2015).

## Concluding Remarks

Emerging evidence indicates that intracellular organelles undergo coordinated changes in shape and/or localization during mitosis. The preliminary steps for these reorganizations begin during G2, when the FAs are selectively dismantled through a mechanism induced by the G2-specific expression of the CDK1/CyclinB complex (Jones et al., 2018) and DEPDC1B, which is a scaffold protein that localizes to FAs, where it inhibits RhoA signaling (Marchesi et al., 2014). The selective dismantling of FAs generates a specific pattern of residual active integrin-based adhesive structures, which could drive the pulling forces that are exerted on MT fibers to direct the repositioning of the centrosome and, as a consequence, of the GC and the nucleus (Champion et al., 2017). These steps are required for cell rounding, which in turn is fundamental for correct spindle formation and orientation. Proper orientation of the division axis is crucial for correct tissue development and the homeostatic processes that maintain the steady state composition and organization of a tissue (Morin and Bellaiche, 2011).

The drastic and rapid modifications of the structure and localization of subcellular organelles during mitosis could be necessary not only to allow their inheritance, but also to remove possible steric interferences with the assembly of the spindle. Furthermore, it is also emerging that the endomembrane machineries have an active role in the formation of the spindle apparatus. The most investigated example is offered by the GC, whose mitotic partitioning can be schematically divided into consecutive steps (Persico et al., 2009). Specifically, the G2-specific unlinking of the Golgi ribbon stimulates the recruitment and activation of Aurora A at the centrosome, which is a necessary step for cell entry into mitosis (Figures 2B,a; Ayala and Colanzi, 2017; Barretta et al., 2016). Then, the disassembly of the Golgi stacks, which occurs after mitosis onset, regulates events that are necessary for the correct formation of the mitotic spindle (Figures 2C,b,c; Wei et al., 2015; Guizzunti and Seemann, 2016). Similar requirements are also emerging for other organelles: the recycling endosomes are relocated around the centrosome to be divided in daughter cells (Figures 2D,c) (Hehnly and Doxsey, 2014); the peroxisomes have to be correctly localized at the spindle to allow its correct orientation (Figures 2D,c) (Kashatus et al., 2011; Asare et al., 2017); and mitochondria fission is necessary for cytokinetic furrow closure (Figures 2E,d; Kashatus et al., 2011).

As a consequence, these events have to be coordinated by signaling pathways. A potential integrator of such signals is Aurora A, which is activated by GC unlinking during G2 to induce centrosome maturation (Barretta et al., 2016), and is also activated by a GM130-based pathway to induce spindle formation after mitosis onset (Wei et al., 2015). In addition, the activity of Aurora A is regulated by FA dismantling, which allows the relocation of some FA-associated scaffolds to the centrosomes, where they interact with Aurora A to protect this kinase from the action of phosphatases (Pugacheva and Golemis, 2006; Pugacheva et al., 2007). Interestingly, Aurora A is also necessary for the fission of mitochondria (Kashatus and Counter, 2011). Then, during mitosis, protein machineries that ordinarily operate at the organelle level are repurposed to direct the proper spindle formation and orientation, and the cleavage of the mitotic furrow (Table 1).

Thus, with this review we highlight that in addition to DNA segregation, the accomplishment of a correct cell division also requires the proper segregation of intracellular organelles, with likely important implications for organism development and tissue homeostasis. Until now, the only evidence of the importance of correct organelle inheritance for organism development has been provided by the effects of peroxisome misplacement, which results in structural alterations of the epidermis (Asare et al., 2017), and of the lack of DEPDC1B expression, which leads to severe defects of zebrafish morphogenesis (Marchesi et al., 2014). We anticipate that additional important functional roles will be revealed when novel strategies to specifically perturb the mitotic inheritance of other organelles will be developed.

## Author Contributions

All authors contributed to the critical reading of the literature and discussion, writing the text, collecting the references, and preparing the figures.

## Conflict of Interest Statement

The authors declare that the research was conducted in the absence of any commercial or financial relationships that could be construed as a potential conflict of interest.
